# Optimization of the Four Most Effective Factors on β-Carotene Production by *Dunaliella salina* Using Response Surface Methodology

**DOI:** 10.22037/ijpr.2019.1100752

**Published:** 2019

**Authors:** Leila Zarandi-Miandoab, Mohammad-Amin Hejazi, Mohammad-Bagher Bagherieh-Najjar, Nader Chaparzadeh

**Affiliations:** a *Department of Biology, Azarbaijan Shahid Madani University, Tabriz, Iran.*; b *Department of Biology, Faculty of Sciences, Golestan University, Gorgan, Iran.*; c *Department of Food Biotechnology, Branch for Northwest & West region, Agricultural Biotechnology Research Institute of Iran, Agricultural Research, Education and Extension Organization (AREEO), Tabriz, Iran.*

**Keywords:** Dunaliella salina, β-Carotene production, Optimization, RSM, Pre adaptation

## Abstract

During recent years, there was growing demand in using microalga valuable products such as β-carotene in health care. β-Carotene has anti-cancer and anti-aging properties for human. In *Dunaliella salina* cells, β-carotene has a major protecting role for biomolecules, when the production of reactive oxygen species is elevated. In the present study, we investigated the influence of the four most effective factors (light intensity, temperature, nitrate and salinity concentration) and their interactions on the β-carotene production and the total chlorophyll/β-carotene ratio in low light adapted *D. salina* cells. Box-Benken design and response surface methodology (RSM) were used for this purpose and optimization of the factor levels. Two models were developed to explain how β-carotene productivity and the total chlorophyll/β-carotene ratio may depend on the stress factors. Among the four stress variables for β-carotene production, light intensity was stronger than the others. Meanwhile, interaction between light intensity and salt concentration exhibited the most important effect on the total chlorophyll/ β-carotene ratio. The predicted optimal conditions for maximum β-carotene productivity and minimum total chlorophyll/β-carotene ratio were derived from the fitted model in 200 µmol photons m^-2^s^-1^ light intensity, 25 ºC, 0.9 mM nitrate and 3.8 M NaCl. When the predicted condition was tested experimentally, the expected results were observed. This suggests that overproduction of β-carotene in *D. salina* under certain conditions depends on used light intensity for preadaptation. The step-wise manner applying of stresses may act as a beneficial strategy to β-carotene overproduction.

## Introduction

There has been a growing interest in modelling approaches for optimization of critical metabolite production from microalgae, in recent years (1-3). Carotenoids are important metabolites, with a variety of functions which comprise a large and diverse group in plants and alga and cover more than 700 different biochemical molecules (4, 5). β-Carotene, the most important carotenoid, is considered as an excellent additive for food and cosmetic industries because of its attractive colour and functional properties. Thereby, universal demands lead to a market value of $261 million in 2010. This market is expected to grow to $334 million by 2018 at a compound annual growth rate of 3.1%. Because of medical effects on vision and heart health and antioxidant, anti-cancer (6), anti-aging, and immunomodulatory properties (7, 8), however; β-carotene have a prominent status in pharmaceutical research. All of these functions depend on the source of β-carotene production. Solely, pure and natural β-carotene is simply digestible and has shown positive effects in the treatment of disorders while synthetic β-carotene not. Natural β-carotene mainly produced by micro-algae and higher plants. *Dunaliella salina* is a unicellular green alga that is known as the only biological source accumulating natural β-carotene approximately 10 - 15% of its body weight. In *D. salina*, β-carotene represent up to 95% of total carotenoids (9). *D. salina* can be adapted to sudden changes in salt concentration, irradiance and nutrient availability in natural habitats (10, 11). The colour of *D. salina* cells changes from green to red under harsh conditions, such as elevated light intensity, high salinity, low nutrient supplies or extreme temperatures, (12, 13). The red *D. salina* cells accumulate more β-carotene in plastid sequestering structures, lipid globules named plastoglobolins (14, 15), in inter thylakoid space of the chloroplast instead of thylakoid membranes (16). Accumulation of β-carotene in plastoglobolins leads to a reduction of the chlorophyll/carotenoid ratio (17). This ability provides *D. salina* as an excellent biological source for commercial development (18). The low chlorophyll content of *D. salina* is an important factor for pure natural β-carotene (with more than 41% 9-cis isomer of β-carotene (19) extraction. The reduction of growth rate by abiotic stresses plays a crucial role in maximizing β-carotene production (20). Most studies regarding to reduction of growth rate and production of β-carotene have been carried out using only one or two factors (light intensity, temperature, nutrient or salt concentrations) at the same time (Table 1).

Closed culture systems compared to open ponds potentially produce higher biomass and carotenoid concentration (19). To reduce the growth rate in industrial closed systems, high intensity of light (11), extreme temperatures (28), and high amount of salt (16) or limitation in nutrients (36) in culture medium must be applied. Two problems must be solved for application of high light intensities. First, digital control for stable temperatures is necessary and second, massive power usage is expensive in industry and large scale production systems. Therefore, finding a new strategy for optimization of β-carotene production under relatively low light irradiations could be remarkably economic. In spite of the large number of studies on this subject, finding a feasible model for optimization of β-carotene production is still controversial. Application of mathematical models for optimization of the fermentation process (30) is a relatively new strategy. Mathematical formulation of algal primary productivity was used since 1995 (37, 38). Response surface methodology (RSM) is a proper technique for modelling and optimizing a response affected by several variables. The aim of this study was to optimize the β-carotene production by various factors including light intensity, temperature, nitrate, and salt concentrations in *D. salina*. For this purpose, a statistical experimental design was employed rather than the one-factor-at-a-time approach. As responses of *D. salina* cells, the rate of β-carotene production and the rate of total chlorophylls/ β-carotene ratio have been measured in the mentioned bioprocess.

## Experimental

*Microalga strain and culture medium*

*D. salina* strain CCAP 19/18 was provided by the branch of Northwest and West region, Agricultural Biotechnology Research of Iran [ABRII NW] (Tabriz). The cultures were grown in modified Johnson medium (27) during the years 2012 and 2013. Different concentrations of NaCl (2, 3 and 4 M) and or KNO_3_ (0, 2.5 and 5 mM) were added to the media. 

*Cultivation conditions*

In order to cultivate algal culture, white compact fluorescent lamps with 145W (Nama‌Nor) were selected as light source (21). The experiments were conducted in two steps. First, in order to prephotoadaptation, *D. salina* was cultured at a light intensity of 50 µmol photon m^-2^s^-1^ and 20 ± 2 ºC for one week (media chemical composition not in limiting rate). Salinity, nitrate concentration, and temperature were selected according to many literatures and this light intensity was selected in order to prephotoadaptation, adapts the cells to low light before exposure to different levels of high lights. Then in a second step, the cells were exposed to combination of stressors for 2 weeks according to RSM designed experiments (Table 3). Nguyen and co-workers also used a two-step method (11). All experiments were done in triplicate in 250 mL Erlenmeyer flasks, containing 150 mL of fresh medium. The average of the initial cell number was 4×10^6^ cells.mL^-1^. 

*Variables measurement*

*Cell count*

The cell number was determined by direct counting. The cells were immobilized and stained by Lugol´s solution and counted using 0.1 mm deep counting chamber (Neubauer) and light microscope (27).

*Pigment analysis*

To measure pigment concentrations, the precisely defined spectrophotometric method was applied. In brief, the pigments were extracted from algal pellets in 80% acetone after removal of cell debris by centrifugation at 8000 rpm (5719×g) for 5 min. Supernatant absorbency was measured at 412, 431, 460, and 480 nm with spectrophotometer (Perkin Elmer precisely-Lambda 35-UV/Vis spectrometer) (39) and pigments content (µg/ml β-carotene) calculated using the following formula. Final data of pigments content present by pg/cell. The sufﬁces *Ca*, *Cb* and *Cc* stand for chlorophyll *a*, chlorophyll *b* and β-carotene, respectively.

*Ca*= – 1.709A412 + 11.970A431 – 2.998A460 – 5.708A480

*Cb*= – 0.171A412 – 0.230A431 + 11.871A460 – 13.248A480

*Cc*= – 0.430A412 + 0.251A431 – 4.376A460 + 13.216A480 

All measurements were performed in three replicates. For calculating the **r**ate of **β**-carotene production per **c**ell (RBC) and the **r**ate of **t**otal chlorophylls/β-carotene per **c**ell (RTC) the slope of the regression line for each response (Rate = *dy/dx*) was chosen (supplementary data 1). *dy* is (*y*_2_*-y*_1_), and *y*_1_ is β-carotene content per cell or total chlorophylls/β-carotene per cell at first day and *y*_2_ is β-carotene content per cell or total chlorophylls/β-carotene per cell at 14^th^ days. *dx* is (*x*_2_*-x*_1_), and *x*_1_ is first day of experiments and *x*_2_ is 14^th^ days of experiments. By this way the rates amounts may show positive or negative value.

*Experimental design and statistical analysis*

Response surface methodology (RSM) was applied to evaluate the effect of the four factors (light intensity, temperature, nitrate, and salt concentration) on β-carotene content of the cells. The factors were studied at the three different levels described in Table 2. Using Box-Behnken design as one of the mostly used response surface methods, a total of 25 experiments were carried out in randomized design 

(Table 3).

The selection of the ranges was based on several previous studies, indicated in Table 1. This design was considered as the suitable design for exploring quadratic response surfaces and constructing second order polynomial models by using the MINITAB16 software. For predicting the optimum point, a second order polynomial function was ﬁtted to correlate the relationship between independent variables and responses. For 4 factors, the corresponding equation is according to equation (1):

(1) *Y* = β_0_ + β_1_*X*_1_ + β_2_*X*_2_ + β_3_*X*_3_ + β_4_*X*_4_ + β_12_*X*_1_*X*_2_ + β_13_*X*_1_*X*_3_ + β_14_*X*_1_*X*_4_+ β_23_*X*_2_*X*_3_+ β_24_*X*_2_*X*_4_+ β_34_*X*_3_*X*_4_+ β_11_*X*^2^_1_ + β_22_*X*^2^_2_ + β_33_*X*^2^_3_+ β_44_*X*^2^_4_

Where Y represents the response variable; β_0_ is a regression coefficient (model constant), β_1_, β_2_, β_3,_ and β_4_ are linear coefﬁcients, and also β_12_, β_13_, β_14_, β_23_, β_24,_ and β_34_ are interaction effect coefﬁcients; β_11_, β_22_, β_33_ and β_44_ are quadratic coefﬁcients, and also *X*_1_*, X*_2_*, X*_3,_ and *X*_4_ are the coded levels of independent variables. The terms *X*_1_*X*_2_ and *X*_2i_ (i = 1, 2, 3 or 4) represent the interaction and quadratic terms, respectively. The significance of the regression coefficients was determined by Students *t*-test. The second order model equation was determined by Fishers test. The accuracy of the model was calculated by the regression coefﬁcients *R*^2^ and adjusted *R*^2^ (Adj *R*^2^). To identify the statistically signiﬁcant terms, the analysis of variance (ANOVA) was employed. These statistical analysis could able us to judge on validity of the model and its reproducibility (40). 

**Table 1:** Summary of the studies on the effect of different abiotic factors on β-carotene production on various species of *Dunaliella*

**Light intensity**	**Variables**			**Organism**	**Year**	**Researchers (Reference)**
	**Temperature**	**Nutrients**	**Salinity**			
*		*	*	*Dunaliella bardawil*	1983	(Ben-Amotz, Avron 1983) (21)
			*	*Dunaliella salina*	1987	(Al-Hasan, Ghannoum *et al. *1987) (22)
*				*Dunaliella bardawil*	1990	(Lers, Biener *et al. *1990) (23)
*				*Dunaliella*	1994	(Vorst, Baard *et al. *1994) (20)
	*			*Dunaliella salina*	1996	(Mendoza, Jimenez Del Rio *et al. *1996) (24)
		*	*	*Dunaliella*	1998	(Marin, Morales *et al. *1998) (25)
*				*Dunaliella viridis*	2001	(Gordillo, Jimenez *et al. *2001) (26)
*				*Dunaliella salina*	2003	(Hejazi, Wijffels 2003) (27)
*	*	*	*	*Dunaliella*	2005	(Dipak 2005) (28)
*		*	*	*Dunaliella salina*	2008	(Coesel, Baumgartner *et al. *2008) (29)
		*		*Dunaliella salina*	2010	(Jesus, Rubens Filho 2010) (30)
		*	*	*Dunaliella salina*	2011	(Pasqualetti, Bernini *et al. *2011) (16)
			*	*Dunaliella tertiolecta*	2011	(Tammam, Fakhry *et al. *2011) (31)
			*	*Dunaliella salina*	2011	(Narvaez-Zapata, Rojas-Herrera *et al. *2011) (10)
			*	*Dunaliella sp.*	2011	(Rad, Aksoz *et al. *2011) (32)
	*			*Dunaliella*	2012	(Ali-zadeh 2012) (33)
		*		*Dunaliella salina*	2013	(Nikookar, Rowhani *et al. *2013) (34)
*				*Dunaliella salina*	2013	(Fu, Guomundsson *et al. *2013) (35)
*		*		*Dunaliella salina*	2013	(Dhanam, Dhandayuthapani 2013) (1)
*		*	*	*Dunaliella salina*	2014	(Fu, Paglia *et al. *2014) (2)

**Table 2:** Process variables and their experimental levels

**Variable**	**Symbol**	**Ranges and levels**		
		**-1**	**0**	**+1**
Light intensity (µmol photons m-2s-1)	*X* *1*	200	600	1000
Temperature (ᵒC)	*X* *2*	25	30	35
Nitrate concentration (mM)	*X* *3*	0	2.5	5
Salt concentration (M)	*X* *4*	2	3	4

**Table 3:** Experimental design matrix and responses based on experimental runs proposed by 4-factors Box-Behnken design. RBC is rate of β-carotene production per cell and RTC is rate of total chlorophylls/β-carotene per cell. Rate were calculated by division of changes in β-carotene amount or total chlorophylls/β-carotene per 14 days during origin and end of experiments Rate = *dy/dx*. Positive and negative amounts show positive or negative rates for each response

Independent variables					RBC				RTC		
**RUN**	**X** **1**	**X** **2**	**X** **3**	**X** **4**	**Mean**	**±**	**SE**	**Mean**		**±**	**SE**
1	200	25	2.5	3	**0.1807**	±	0.0105	-0.0156		±	0.0074
2	1000	25	2.5	3	-0.0909	±	0.0073	0.0042		±	0.0008
3	200	35	2.5	3	0.0368	±	0.0119	0.0087		±	0.0030
4	1000	35	2.5	3	-0.0131	±	0.0021	-0.1260		±	0.0168
5	600	30	0	2	-0.0235	±	0.0047	-0.0660		±	0.0070
6	600	30	5	2	-0.0120	±	0.0106	-0.0558		±	0.0090
7	600	30	0	4	0.0258	±	0.0090	-0.0723		±	0.0074
8	600	30	2.5	3	0.0286	±	0.0041	-0.0654		±	0.0061
9	600	30	5	4	-0.0723	±	0.0023	-0.0591		±	0.0036
10	200	30	2.5	2	0.1410	±	0.0180	0.0597		±	0.0067
11	1000	30	2.5	2	-0.0062	±	0.0012	-0.2179		±	0.0061
12	200	30	2.5	4	0.1324	±	0.0170	-0.0823		±	0.0032
13	1000	30	2.5	4	-0.0144	±	0.0084	0.0566		±	0.0067
14	600	25	0	3	-0.0035	±	0.0006	-0.0280		±	0.0090
15	600	35	0	3	-0.0494	±	0.0046	-0.0886		±	0.0015
16	600	25	5	3	0.0075	±	0.0013	-0.0284		±	0.0053
17	600	35	5	3	-0.0320	±	0.0101	-0.0578		±	0.0058
18	200	30	0	3	0.1243	±	0.0101	0.0378		±	0.0030
19	1000	30	0	3	-0.0095	±	0.0026	-0.1378		±	0.0222
20	200	30	5	3	0.1241	±	0.0114	-0.0429		±	0.0070
21	1000	30	5	3	-0.0404	±	0.0087	-0.0449		±	0.0066
22	600	25	2.5	2	-0.0234	±	0.0045	-0.0373		±	0.0088
23	600	35	2.5	2	-0.0075	±	0.0011	-0.0465		±	0.0017
24	600	25	2.5	4	-0.0110	±	0.0022	-0.0597		±	0.0033
25	600	35	2.5	4	-0.1050	±	0.0072	-0.0519		±	0.0022

X_1_: Light intensity (µmol photons m^-2^s^-1^) X_2_: Temperature (^ᵒ^C) X_3_: Nitrate concentration (mM) X_4_: Salt concentration (M)

**Table 4:** Analysis of variance (ANOVA) for response surface quadratic models

**Source of variance**	**Response RBC**					**Response RTC**				
	**Sum of squares**	**Degree of freedom**	**Adjusted mean square**	**F-value**	**P**	**Sum of squares**	**Degree of freedom**	**Adjusted mean square**	**F-value**	**P**
Regression	0.386	14	0.027	**78.76**	**0.000**	0.242	14	0.0173	**38.64**	**0.000**
L	0.208	1	0.208	**595.67**	**0.000**	0.046	1	0.046	**103.67**	**0.000**
T	0.013	1	0.013	**37.61**	**0.000**	0.009	1	0.009	**21.74**	**0.000**
N	0.002	1	0.002	**5.69**	**0.020**	0.001	1	0.001	2.43	0.123
S	0.003	1	0.003	**9.08**	**0.004**	0.002	1	0.002	**5.05**	**0.028**
L^2^	0.074	1	0.030	**87.62**	**0.000**	0.005	1	0.007	**17.59**	**0.000**
T^2^	0.016	1	0.026	**75.49**	**0.000**	0.005	1	0.005	**12.75**	**0.001**
N^2^	0.003	1	0.007	**21.93**	**0.000**	0.000	1	0.000	0.65	0.421
S^2^	0.009	1	0.009	**25.87**	**0.000**	0.000	1	0.000	1.15	0.288
LT	0.036	1	0.036	**105.25**	**0.000**	0.017	1	0.017	**39.97**	**0.000**
LN	0.000	1	0.000	2.03	0.159	0.022	1	0.022	**50.51**	**0.000**
LS	0.000	1	0.000	0.00	0.983	0.130	1	0.130	**290.55**	**0.000**
TN	0.000	1	0.000	0.09	0.769	0.000	1	0.000	1.63	0.207
TS	0.009	1	0.009	**25.81**	**0.000**	0.000	1	0.000	0.49	0.488
NS	0.009	1	0.009	**25.72**	**0.000**	0.000	1	0.000	0.02	0.902
Residual error	0.023	66	0.000			0.029	66	0.0004		
Pure Error	0.010	56	0.000			0.011	56	0.000		
Total	0.409	80				0.271	80			
R^2^	**94.35%**					**89.13%**				
R^2^ adjusted	**93.15%**					**86.82%**				

**Table 5:** Obtained optimum values of the process variables and responses

	Independent Variables				**Response RBC**		**Response RTC**	
	**X1**	**X2**	**X3**	**X4**	**Experimental value**	**Predicted value**	**Experimental value**	**Predicted value**
**Optimum point**	200	25	0.9	3.8	0.190 ± 0.012	0.191	-0.0626±0.0024	-0.0608

**Figure 1:**
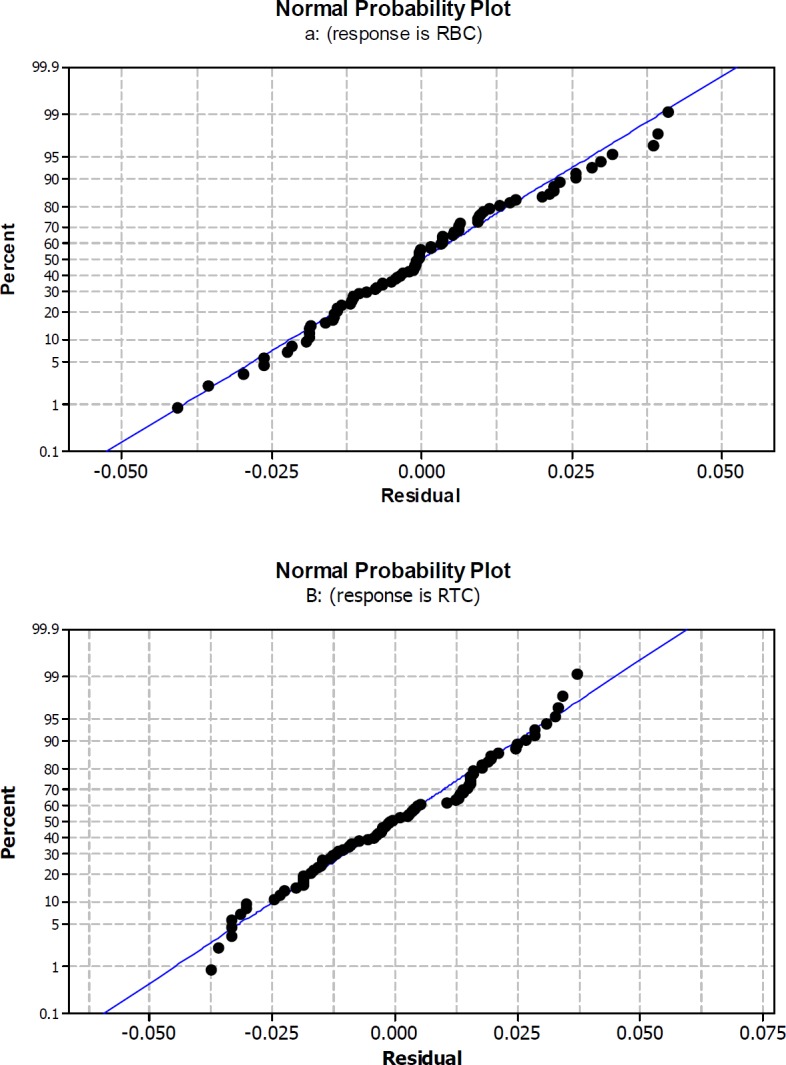
Normal probability plot for rate of β-carotene production per cell RBC (a) and rate of total chlorophylls/β-carotene per cell RTC (b) Rate were calculated by division of changes in β-carotene amount or total chlorophylls/β-carotene per 14 days during origin and end of experiments Rate = *dy/dx*

**Figure 2:**
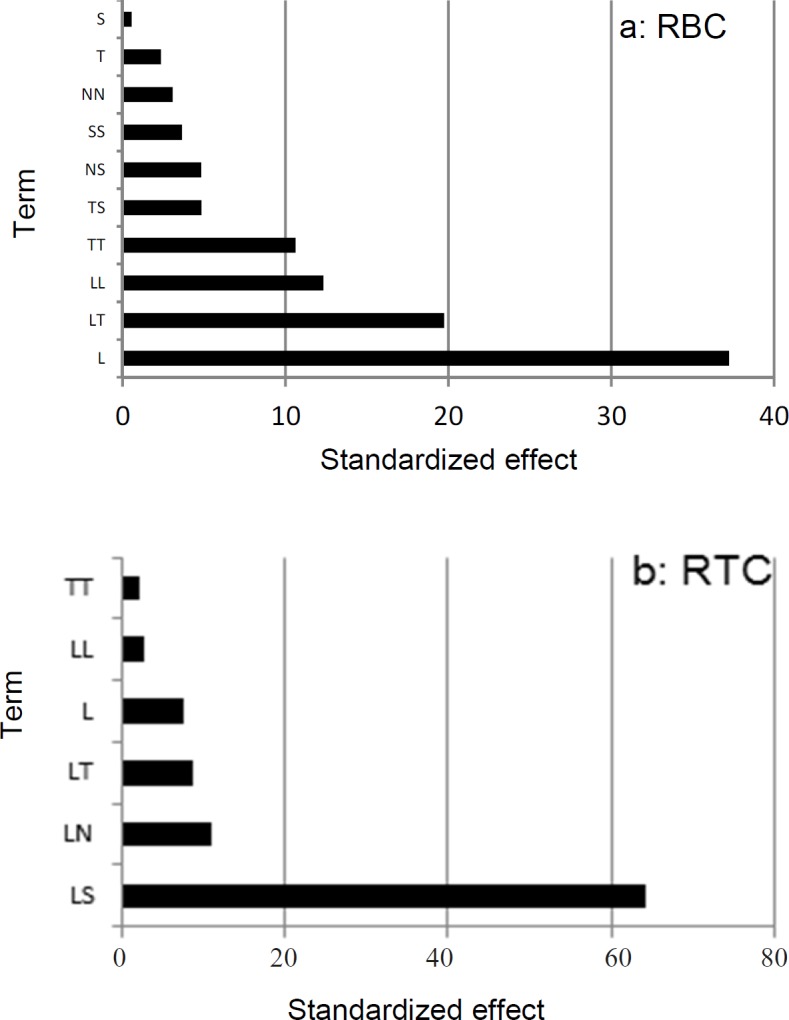
Pareto chart for rate of β-carotene production per cell RBC (a) and rate of total chlorophylls/β-carotene per cell RTC (b) Pareto values calculated using *Pi*=$\left(\frac{bi^{2}}{\sum bi^{2}}\right)\times 100$ , (i ≠ 0) Where b is the related regression coefﬁcient of the factor

**Figure 3:**
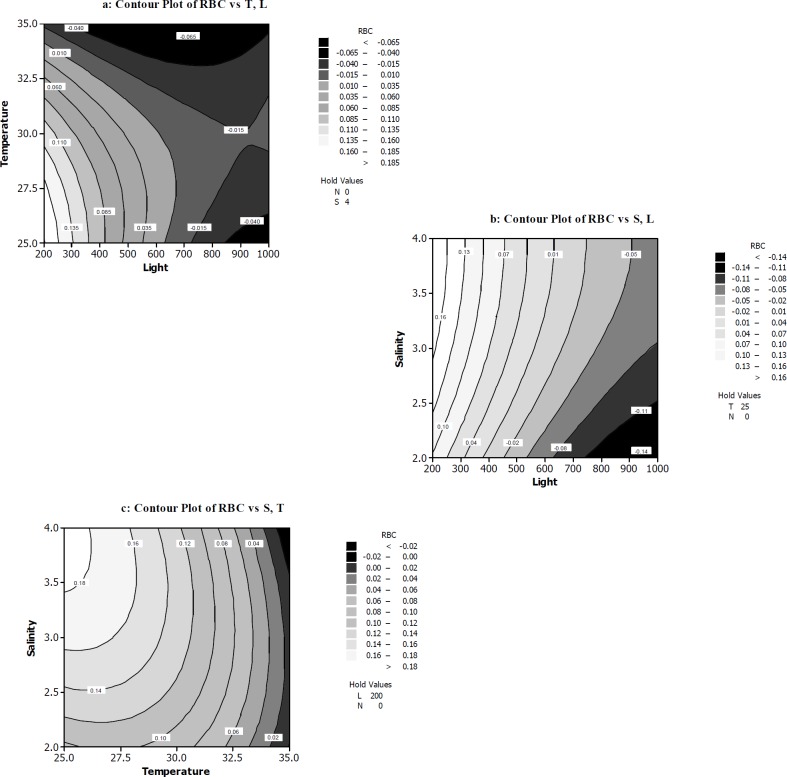
The response surface and contour plots of rate of β-carotene production per cell RBC **(a) **The function of temperature (^ᵒ^C) and light intensity (µmol photons m^-2^s^-1^) on RBC. **(b)** The function of light intensity (µmol photons m^-2^s^-1^) and salt concentration (M NaCl) on RBC. **(c)** The function of temperature (^ᵒ^C) and salt concentration (M NaCl) on RBC

**Figure 4:**
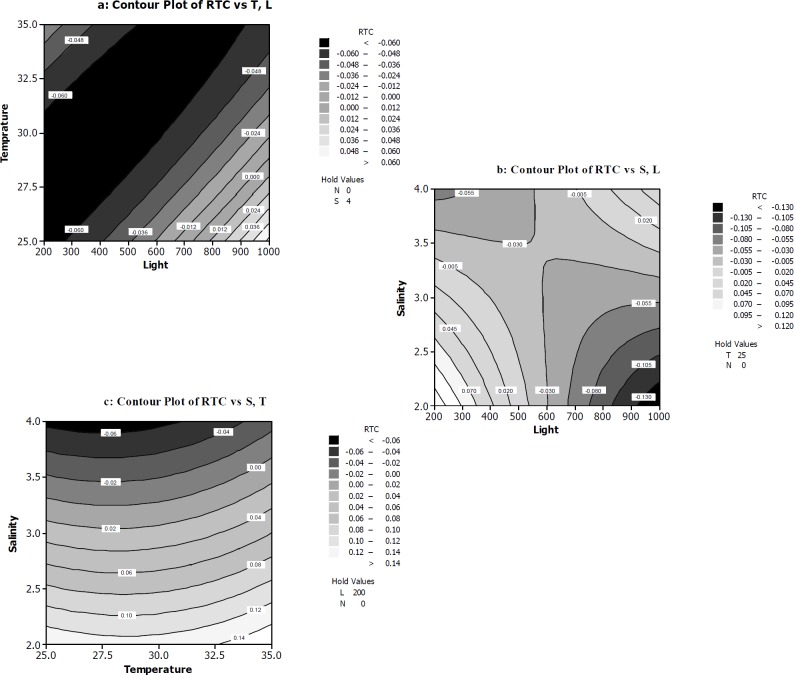
The response surface and contour plots of rate of total chlorophylls/β-carotene per cell RTC **(a) **The function of temperature (^ᵒ^C) and light intensity (µmol photons m^-2^s^-1^) on RTC. **(b)** The function of light intensity (µmol photons m^-2^s^-1^) and salt concentration (M NaCl) on RTC. **(c)** The function of temperature (^ᵒ^C) and salt concentration (M NaCl) on RTC

**Figure 5:**
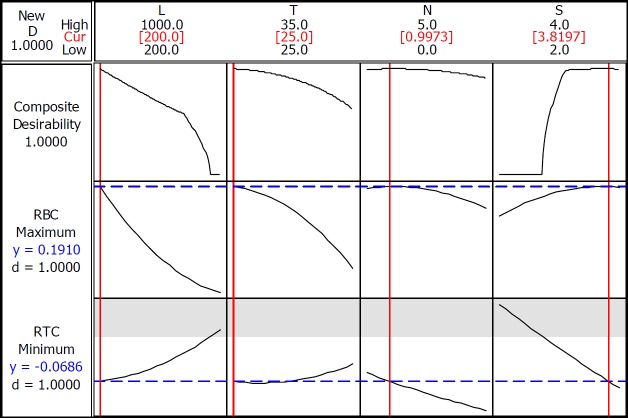
Optimality plot to locate optimum factor levels for maximizing rate of β-carotene production per cell **(RBC)** and minimizing rate of total chlorophylls/β-carotene per cell** (RTC)**

All used statistics were based on a conﬁdence level of 95%, so *p* ≤ 0.05 was considered to indicate a statistically signiﬁcant difference and also used to show the power of the signiﬁcance. For further interpretation of the obtained results, the Pareto analysis was performed. Using this analysis, the percentage effect of each factor on the responses can be calculated according to the following relationship (30): 

*Pi*=$\left(\frac{bi^{2}}{\sum bi^{2}}\right)\times 100,(i\neq 0)$

Where b is the related regression coefﬁcient of the factor. 

## Results and Discussion

*Box-Behnken Model Analysis*

For the first time RSM was used to optimize two responses under four independent factors. In the present study, light intensity, temperature, nitrate, and salt concentrations were considered as the independent process variables and their individual and interactive effects on RBC and RTC (as responses) were investigated using the Box-Behnken design approach and the data are presented in Table 3. With respect to data values in Table 3 maximum positive RBC was achieved in experiment 1 while minimum negative RTC was achieved in experiment 11. The statistical significance of the Box-Behnken models were evaluated by the ANOVA test and the results were illustrated in Table 4 with *R*^2^ and adjusted *R*^2^ amounts. Interaction coefficient of LT, TS, and NS for RBC and alo LT, LN, and LS for RTC are significant at the same confidence level. In order to improve models, the insignificant model terms were omitted from quadratic equation. This resulted in following polynomial equation (2) and (3) based on the coded levels for RBC and RTC. 

(2)** RBC** = 0. 032064 -0.076143 *L* -0.019132 * T* -0.007439 *N* -0.009401 *S* +0.055438 *LT* -0.027451 *TS* -0.027406 *NS *+0.043806 *L*^2^ -0.040659 *T*^2^ -0.021915 *N*^2^ -0.023804 *S*^2^

(3) **RTC** = -0.073514 -0.035914 *L* -0.016447 *T* +0.007923 *S* -0.038627 *LT* +0.043422 *LN* +0.104140 *LS* +0.022192 *L*^2^ +0.018893 *T*^2^

The *R*^2^ value for RBC and RTC models, indicate that the relationship between the variables and responses was good depicted by second order models. *R*^2^ values indicate a high correlation between experimental and predicted values for both responses (Figure 1 a and b). In a system with different number of independent variables, adjusted *R*^2^ (Adj- *R*^2^) is more suitable for evaluating the model goodness of fit (41). According to the current results Adj- *R*^2^ values (93.15% and 86.82% for RBC and RTC, respectively) were close to the corresponding *R*^2^ values (94.35% and 89.13% for RBC and RTC, respectively).

*Screening of Main Effects*

To visualize the importance of each factor in full quadratic models and to sort out which effect exerts a signiﬁcant inﬂuence, the Pareto value was calculated and shown in Figure 2 indicating that the most important factor in RBC was light intensity (Pareto amount = 37.24%). For RTC the interaction between light intensity and salt concentration exhibited the most important effect (Pareto amount = 64.34%). These data suggest that light is the most important factor for both RBC and RTC responses.

*Effect of Variables on Rate of Β-Carotene Production per Cell*

Current knowledge about the interaction of salinity, low nutrient levels, high temperatures and high irradiance on β-carotene production by *D. salina* is scared. Then, we tried to optimize pure β-carotene production in this microalga under combined sever conditions, after preadaptation stage for growth. To study the interaction of all four variables on RBC, two dimensional contours were plotted keeping two variables constant at a certain level and the other two variables within the experimental ranges. As seen in Figure 3a, the maximum of RBC occurred when light intensity (200-250 µmol photons m^-2^s^-1^) and temperature (25-27.5 ºC) were at their minimum levels, while the nitrate (0mM) and salt concentration (4M) was kept at the minimum and maximum level, respectively. Also in the RBC polynomial equation resulted from our experiments, the light intensity and temperature exhibited considerable negative effects on RBC.

ANOVA Table (Table 4) and Pareto chart (Figure 2) confirm the significant impact of these two variables on RBC. Figure 3b indicate that high light intensities can slightly increase RBC, while salt concentration was 3-4 M. Figure 3b contour again shows the significant impact of light intensities on RBC. This confirms the results acquired from Figure 3a contour. Moreover, Figure 3c shows the temperature of 25-26 ºC and salt concentration of 3.5-4 M enhanced RBC response when light intensity and nitrate concentration were kept at the minimum level (0 mM). In the present study, salinity showed significant effect on RBC. The results illustrated in Table 4 clearly show this claim. Also, in experiments of 1, 10, 12, 18, and 20 in Table 3 the β-carotene production rate was maximum and varied between 0.12 and 0.18. For example the highest RBC occurred in 200 µmol photons m^-2^s^-1^ light intensity, 25 ºC and 2.5 mM nitrate concentration and 3M salt concentration condition (run1).

Furthermore, these data show that the adapted cells to low light intensities (about 50 µmol photons m^-2^s^-1^) when exposed to relatively high light intensities about 200-250 µmol photons m^-2^s^-1^ had much more β-carotene production per cell. This finding was confirmed by other scientists (42-45, 28). Of course, it must be mentioned that our results about adopted cells to low light is a little different from previous data. Whereas Xu and co-workers (46) pointed to differences in *Dunaliella *isolates in this case.

*Effect of Variables on Rate of Total Chlorophylls/ β -Carotene per Cell*

Biosynthesis of carotenoids is a complex process which is coordinated with the biogenesis of chlorophylls and proteins of the photosynthetic apparatus (47). From this point of view, not only over production of β-carotene per cell is very important, but also its purity from other lipophilic molecules such as chlorophylls that could be co-extracted with β-carotene is important too. Hence, in the present study, the rate of total chlorophyll/ β-carotene was calculated.

The influence of the variables on RTC was illustrated in Figure 4 (a-c).

Figure 4a shows the RTC decreases in 200-900 µmol photons m^-2^s^-1^ light intensity range and 25-35 ºC temperature range. The polynomial equation of RTC indicated negative effect of light and temperature on RTC. Also ANOVA table confirmed the significance of light and temperature and salt concentration at *p* ≤ 0.05 on RTC. Two regions of plot illustrate minimum amounts of RTC (Figure 4b). It was evident that at the first region, high level of salt concentration combined with relatively high level of light intensity was able to reduce RTC. While in the second region, low concentration of salt and very high light irradiation led to a decrease in RTC amounts. Interestingly, in spite of this fact that RTC in second region is smaller than RTC at first region, the authors believe that reaching a minimum amount of RTC by increasing salt concentration in culture medium is better than increasing light intensity. The interaction effect of light and salt concentration has a positive effect on RTC as indicated by the ANOVA analysis and the polynomial equation of RTC. Figure 4c illustrates that high salt concentration at 25-30 ºC can decrease RTC when light intensity was constant at relatively high about 200 µmol photons m^-2^s^-1^. In all contours of Figure 4 nitrate concentration was kept at low level (0 mM).

Thus, we can say that when algal culture was transferred from low light (50 µmol photons m^-2^s^-1^) to relatively high light (200 µmol photons m^-2^s^-1^), β-carotene production key in *D. salina* cell factory turn ON and at the same time chlorophyll degradation increased. Our interpretation is supported by Pirastru and his team believed the changes in the algal physiological state induced by intense conditions (for example 200 µmol photons m^-2^s^-1^ irradiance) (48) lead to changes in the activity in photosynthetic apparatus. These processes finally lead to the synthesis and accumulation of carotenoids. But, if the cells have to undergo higher light intensities such as 600 or 1000 µmol photons m^-2^s^-1^ after adaptation to low lights, they need to apply other ways to protect them and save viability except pigment response. 

On the other hand, thereby β-carotene is a lipophilic high value compound and the low level of chlorophyll can be essential and very important in β-carotene purification, from the economic and industrial point of view, increasing the β-carotene production has a contrary relationship with total chlorophyll/ β-carotene ratio. 

*Finding Optimum Conditions for Maximizing RBC and Minimizing RTC*

Many investigators have recently turned to find an optimum condition for maximum production using optimization tools. This study aimed to examine this method in the living organism of *D. salina* and the metabolic product of β-carotene. The experimental data were fitted into a full quadratic polynomial model for 4 independent variables. The optimization process consists of ﬁnding the combination of input variable settings that jointly optimize the response. Minitab software calculates an optimal solution and draws a plot (Figure 5), which helps to interactively change the input variable settings to perform sensitivity analysis and possibly improve the initial solution.

There are a few reports on the optimization of two related responses. Therefore, we used the quadratic model to predict the optimal conditions for β-carotene maximum production as well as minimum total chlorophyll/ β-carotene ratio. Since maximizing RBC was our priority, we decided to change weight and import values about 9 and 1 for RBC versus RTC, respectively. Surprisingly, when maximizing the RBC and minimizing RTC are considered for optimization, an optimum point of light intensity was introduced at 200 µmol photons m^-2^s^-1^, 25 ºC and 0.9 mM nitrate concentration in culture medium and 3.8 M of salt concentration. Figure 5, optimality demonstrated the plot to locate optimum factor levels for maximizing RBC and minimizing RTC. Based on this prediction and to confirm the adequacy model, the additional experiments were performed at optimum point and the results were showed in Table 5. These values were according to predicted responses and validate the findings of response surface optimization. Therefore, this observation shows that our models have feasible results.

## Conclusion

Traditional optimization tools are very expensive and time consuming, and they cannot also clarify the factual interactions of the parameters of the experimental data and thus lead to misunderstanding of results that are used to choose the significant factors that influenced the process. A statistical approach in experimental design of biotechnological processes is confirmed to overcome the limitations of conventional optimization process and allows quick identification of the important factors and interactions between them. In the current work, the statistical methodology, the Box-Behnken design under RSM is employed in selecting the statistically significant variables and finding the optimal condition of those variables for maximizing pure natural β-carotene production by *D. salina* in a biological process. The present work is the first to report on the application of Box-Behnken design and Response surface methodology for the optimization of pure natural β-carotene from *D. salina*. This study recommends a new guideline for applying stress in a step-wise manner in order to acquire rational pure natural β-carotene production. The result of optimization showed a significant increase in β-carotene content per cell whereas chlorophyll content per cell decreased in relatively high light (200 µmol photons m^-2^s^-1^) at 25 ºC. It can be due to preadaptation growth stage under low light (50 µmol photons m^-2^s^-1^). Thus, to achieve a significant amount of pure β-carotene, we need to use low light intensities for growth and acquire adequate amount of cells and then transfer adapted cells to new culture condition containing high salinity and limited amount of nitrate under relatively high light intensity. These results are considerably different from previous findings. It can be concluded that we can induce light stress without using high light intensities. Our results indicate the optimized condition might result in a major reduction in the cost of pure natural β-carotene production and extraction. Additionally, the current results indicated that the experimental design worked in this project was a good mathematical tool for optimization of β-carotene production and quality of extracted β-carotene from *D. salina*.
